# A prospective interventional cohort study of 175 patients treated by the SpineCor orthosis, following the Scoliosis Research Society Criteria

**DOI:** 10.1186/1748-7161-8-S1-O47

**Published:** 2013-06-03

**Authors:** C Coillard, A Circo, CH Rivard

**Affiliations:** 1 Ste-Justine Hospital, Montreal, Canada

## Background

The mainstay of the conservative treatment still remains the orthosis, which was demonstrated to provide a reduction of curve progression, possibly a decrease in the need for surgery, and sometimes a correction of the existing deformity. The effectiveness of the SpineCor orthosis compared with the natural history of the disease has already been shown for milder and moderate curves[1].

## Aim

To provide confirmation on the demonstrated effectiveness of the Dynamic SpineCor orthosis for adolescent idiopathic scoliosis, following the standardized criteria proposed by the SRS Committee on Bracing and Nonoperative Management[2], and to confirm the stability of the results two years after the end of the treatment.

## Method

From 1993 to 2011, 390 patients treated using the SpineCor orthosis respected the criteria for inclusion recommended by the SRS committee. 198 have a definitive outcome, and 175 have at least 2 years of follow-up.

Assessment of brace effectiveness included; 1) percentage of patients who have 5 degree or less curve progression, and the percentage of patients who have 6 degree or more progression at skeletal maturity, 2) percentage of patients who have had surgery recommended/undergone before skeletal maturity, 3) percentage of patients with curves exceeding 45 degree at maturity (end of treatment) and 4) 2-years follow-up beyond maturity to determine the percentage of patients who subsequently underwent surgery.

## Results

At two years post skeletal maturity, successful treatment (correction >5 degree or stabilization ±5 degree) was achieved in 100 patients of the 175 patients (57.2%) from the time of the fitting of the SpineCor orthosis to the 2 years follow-up point. 41 immature patients (23.4 %) required surgical fusion.(34 while receiving treatment and 5 in the follow-up period).

## Conclusions

The SpineCor orthosis is effective for the treatment of adolescent idiopathic scoliosis. Positive outcomes are maintained after the weaning of the orthosis, since 86.1% of the patients stabilized or corrected their Cobb angle. Moreover, out of the 86.1%, 11.7 % of the patients still had correction of their Cobb angle 2 years after the end of the treatment.
